# AP3×ML3 reduction quality classification for femoral trochanteric fractures: validation for reliability focusing on positive medial cortical support

**DOI:** 10.1186/s13018-023-03555-5

**Published:** 2023-01-24

**Authors:** Junya Itou, Yujiro Kuramitsu, Satoshi Hatta, Ken Okazaki

**Affiliations:** 1 Department of Orthopaedic Surgery, Tokyo Women’s Medical University, 8-1 Kawada-Cho, Shinjuku-Ku, Tokyo, 162-8666 Japan; 2 Department of Orthopaedic Surgery, Funabashi General Hospital, 1-13-1 Kitamoto-Cho, Funabashi, Chiba 273-0864 Japan

**Keywords:** Reduction quality, Femoral trochanteric fractures, Positive medial cortical support, AP3 × ML3 classification, Baumgaertner reduction quality criteria, Mechanical complications

## Abstract

**Introduction:**

This study evaluated the validity of the AP3 × ML3 reduction quality classification, which applies the concept of positive medial cortical support.

**Methods:**

A total of 120 trochanteric fractures classified as AO Foundation/Orthopedic Trauma Association 31A1 and A2 were retrospectively analyzed. The validity of the AP3 × ML3 classification was evaluated by comparison with the Baumgaertner reduction quality criteria. When using the AP3 × ML3 classification, reduction quality was divided into three classes based on the degree of anterior cortical contact between the proximal and distal fragments. Reduction quality was also divided into three classes when using the Baumgaertner criteria. The frequency of mechanical complications, including cut­out, delayed union, and excessive migration of the lag screw, was retrospectively assessed. Intra-observer and inter-observer reliability was assessed using the intraclass correlation coefficient (ICC).

**Results:**

Mechanical complications included 4 cases of cutout (3.3%) and 1 of delayed union (0.8%). Mechanical complications occurred for all levels of reduction quality in both classifications, except for the acceptable of the Baumgaertner criteria. When reduction quality was rated as good, acceptable, and poor the incidence of mechanical complications was 2.5%, 5.7%, and 16%, respectively, under the AP3 × ML3 classification and 3.3%, 0%, and 15.0%, respectively, under the Baumgaertner criteria. The ICC was 0.80 for intra-observer reliability and 0.57 for inter-observer reliability when using the AP3 × ML3 classification and 0.85 and 0.34, respectively, when using the Baumgaertner criteria.

**Conclusion:**

The AP3 × ML3 classification was reliable and easy to use compared with the widely used Baumgaertner reduction quality criteria.

**Level of evidence** 4

## Introduction

**Fig. 1:**
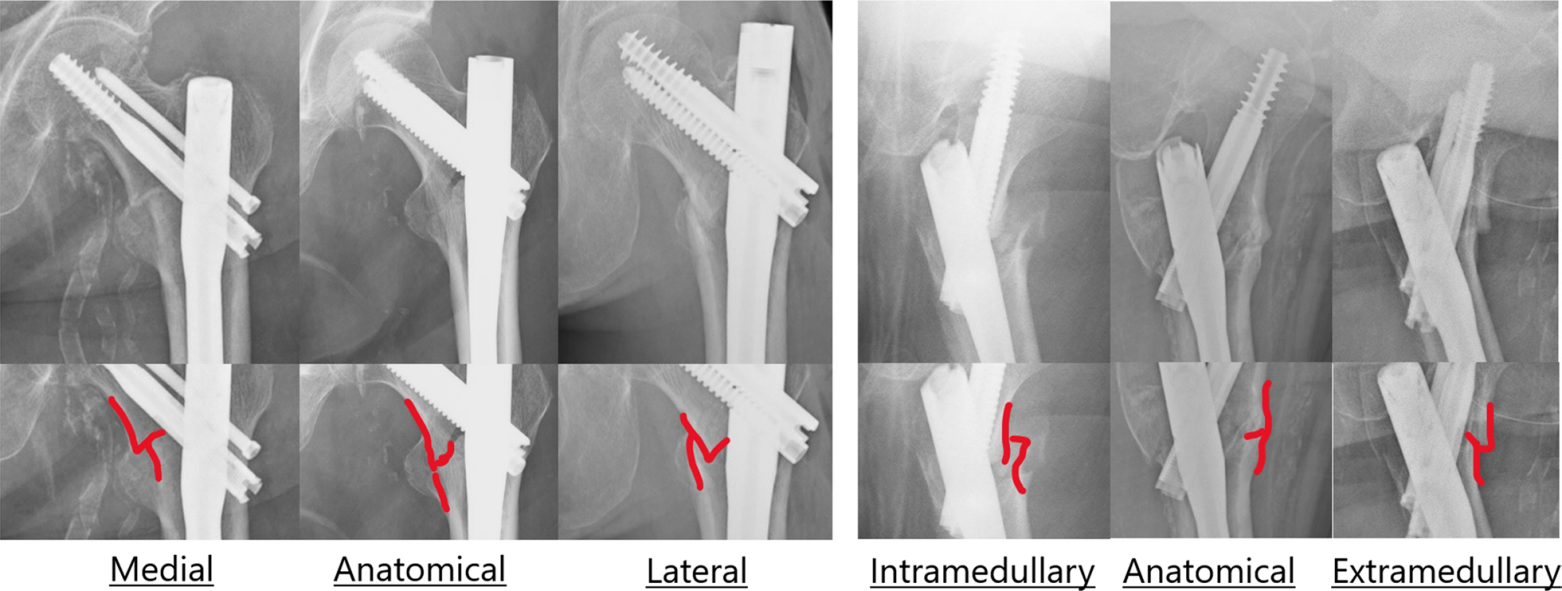
AP3 × ML3 classification

Femoral trochanteric fractures are an important issue for orthopedic surgeons and are increasing in frequency with population aging [1]. Optimal reduction is necessary to achieve good outcomes [2, 3], but the criteria used to assess the reduction quality of trochanteric fractures vary in the literature [4–6]. The criteria most widely used are those devised by Baumgaertner et al. [5, 7, 8] that emphasize anatomical reduction. Recently, the concept of positive medial cortical support (PMCS) has been proposed [2]. PMCS is defined as slight medial displacement of the cortical bone on the side of the proximal fragment and could provide cortical support between the proximal and distal fragments, thereby resisting further sliding of the proximal fragment [9, 10]. A new reduction quality classification for trochanteric fractures, which applies the concept of PMCS, was proposed by Fukuda et al. [11, 12] (Fig. 1). Using their system, anteroposterior (AP) and mediolateral (ML) views are used to categorize three reduction positions. Reduction quality would be more easily classified by this method than by the Baumgaertner criteria because there is no need to measure the neck–shaft angle or the degree of displacement of any fragments. This new classification, known as the AP3 × ML3 classification [12], has not yet been compared with other classification systems or evaluated for validity.

The aim of this study was to evaluate validity of AP3 × ML3 classification by examining the hypothesis that the AP3 × ML3 classification would be useful and reliable compared with the Baumgaertner reduction quality criteria.

## Materials and methods

### Study population

This retrospective study examined all 128 consecutive femoral trochanteric fractures with AO Foundation/Orthopedic Trauma Association (AO/OTA) type 31A1 or A2 femoral trochanteric fractures treated at two institutions between January 2017 and December 2020. Eight fractures that were followed up for less than 3 months after surgery were excluded. The remaining 120 trochanteric fractures (in 117 patients) were included (Table 1). Of these, 49 fractures were treated at institution 1 and 71 fractures were treated at institution 2. The study population included 33 men and 84 women with mean age at surgery of 83.7 ± 9.8 years. Of these fractures, 113 were treated using a cephalomedullary nail and 7 were treated using a sliding hip screw. Whether a cephalomedullary nail or sliding hip screw was used depended on the stability of the fracture and was left to the surgeon’s discretion. Four types of cephalomedullary nail were used during the study period: the InterTAN (Smith & Nephew, Memphis, TN), Gamma3 (Stryker, Kalamazoo, MI), Targon PFT (B. Braun Aesculap, Tuttlingen, Germany), and Unicorn plus (HOYA Technosurgical Corporation, Tokyo, Japan). The two surgical centers worked closely together and followed the same treatment plan. All surgeries were performed by experienced surgeons.

**Table 1:** Demographic and clinical data for 117 patients with 120 trochanteric fractures

Mean age (years)	83.7 ± 9.8
Sex (M/F)	33/84
Implant	Cephalomedullary nail (*n* = 113), sliding hip screw (*n* = 7)
AO/OTA classification	A1, 59; A2, 61
Position of lag screw	Good, 106; poor, 14

*AO/OTA* AO Foundation/Orthopedic Trauma Association

The study was approved by the ethics committees of each participating institution [approval numbers (both institutions): 5659]. Informed consent was obtained via an opt-out procedure.

### Radiological evaluation

The AP3 × ML3 classification and Baumgaertner criteria were compared using lateral AP and ML radiographs obtained in the standard way [4] and retrieved from the picture archiving and communication system. Using the AP3 × ML3 classification (Fig. 1), reduction quality was defined according to the degree of contact between the medial cortex of the proximal fragment and that of the distal fragment as follows: (1) anatomical, medial, or lateral in the AP view; (2) anatomical, intramedullary, or extramedullary in the ML view [11]. Fukuda et al. considered that the risk of postoperative dislocation or cut-out would be greater with the AP lateral type and ML intramedullary type. Therefore, reduction quality was divided into three classes: good when neither type was present, acceptable when one or the other type was present, and poor when both types were present.

When using the Baumgaertner criteria, reduction quality was classified as good, acceptable, or poor (Table 2) as in previous reports [4, 13].

**Table 2:** Baumgaertner reduction quality criteria

Alignment	Anteroposterior view: normal or slight valgus neck–shaft angle*
	Lateral view: less than 20° of angulation
Displacement	Anteroposterior view: less than 4 mm of displacement of any fragment
	Lateral view: less than 4 mm of displacement of any fragment
Quality of reduction	Good: both criteria met
	Acceptable: only one criterion met
	Poor: neither criterion met

*Slight valgus refers to valgus of no more than 10°

Fractures were classified according to the AO/OTA system, and the position of the lag screw was classified as good or poor as in an earlier study [14]. A good position was defined as the lag screw being placed in the center or inferior third of the femoral head.

### Clinical outcomes

Complications occurring up to the final follow-up visit were assessed retrospectively by review of the medical records. Mechanical complications included cut­out, delayed union, and excessive migration of the lag screw. Clinical complications included infection, avascular necrosis, and fatal venous thromboembolism.

### Statistical analysis

All statistical analyses were performed using JMP software (SAS Institute Inc., Cary, NC). A *p* value of 0.05 was considered statistically significant. Continuous variables are reported as the mean (range). Independent-samples *t* tests were performed for all continuous variables and the chi-squared test or Fisher’s exact test for all categorical variables. Stepwise logistic regression analysis was performed to detect factors associated with mechanical complications. The following categorical variables were investigated as predictors of outcome: male sex, poor reduction according to AP3 × ML3 classification, poor reduction according to Baumgaertner criteria, poor positioning of the lag screw, and AO/OTA type A2.

Intra-observer reliability was measured for both the AP3 × ML3 classification and Baumgaertner criteria on two occasions separated by an interval of 7 days for each in 36 randomly selected patients. Inter-observer reliability was evaluated for two observers (JI, YK), each of whom worked independently. As in a previous study [15], the intraclass correlation coefficients (ICCs) value was rated as poor (< 0.5), moderate (0.5–0.75), good (0.75–0.9), or excellent (> 0.9).

## Results

Mechanical complications occurred in 4 cases of cutout (3.3%) and 1 of delayed union (0.8%) (Table 3). All these cases were treated with a cephalomedullary nail and converted to total hip arthroplasty. The average time of cutout was 19.7 weeks (range 3−32 weeks). There were 2 cases of clinical complications: 1 case (0.8%) of late surgical site infection that developed 2 months postoperatively and required additional surgery for removing hardware material plus oral antibiotics and 1 case (0.8%) of avascular necrosis that occurred 9 months postoperatively in at patients with a stable (AO/OTA-A1) fracture and a well-placed implant. There were no cases of fatal venous thromboembolism. In addition, the rate of mechanical complications was comparable between the two institutions (2.8% vs. 6.1%;* p* = 0.39). The average time of follow-up was 13.2 months (range 3−49 months).

**Table 3:** Details of cases with mechanical complications

Case No.	Complication	AP3 × ML3	Baumgaertner	Position of lag screw	AO/OTA classification
1	Cutout	Acceptable	Poor	Good	A2
2	Cutout	Poor	Poor	Poor	A1
3	Cutout	Good	Good	Poor	A2
4	Cutout	Good	Good	Poor	A1
5	Delayed union	Acceptable	Poor	Poor	A2

*AP3 × ML3* AP3 × ML3 classification, *Baumgaertner* Baumgaertner reduction quality criteria, *AO/OTA* AO Foundation/Orthopedic Trauma Association

Table 4 shows the results of the intra-observer and inter-observer reliability analysis. The intra-observer ICC was 0.80, and the inter-observer ICC was 0.57 when using the AP3 × ML3 classification and 0.85 and 0.34, respectively, when using the Baumgaertner criteria.

**Table 4:** Reliability of each reduction classification

Variable	ICC	Reliability
AP3 × ML3 (1.1)	0.80	Good
Baumgaertner (1.1)	0.85	Good
AP3 × ML3 (2.1)	0.57	Moderate
Baumgaertner (2.1)	0.34	Poor

1.1 indicates intra-observer reliability and 2.1 indicates inter-observer reliability

*AP3 × ML3* AP3 × ML3 classification, *Baumgaertner* Baumgaertner reduction quality criteria, *ICC* intraclass coefficient

**Fig. 2:**
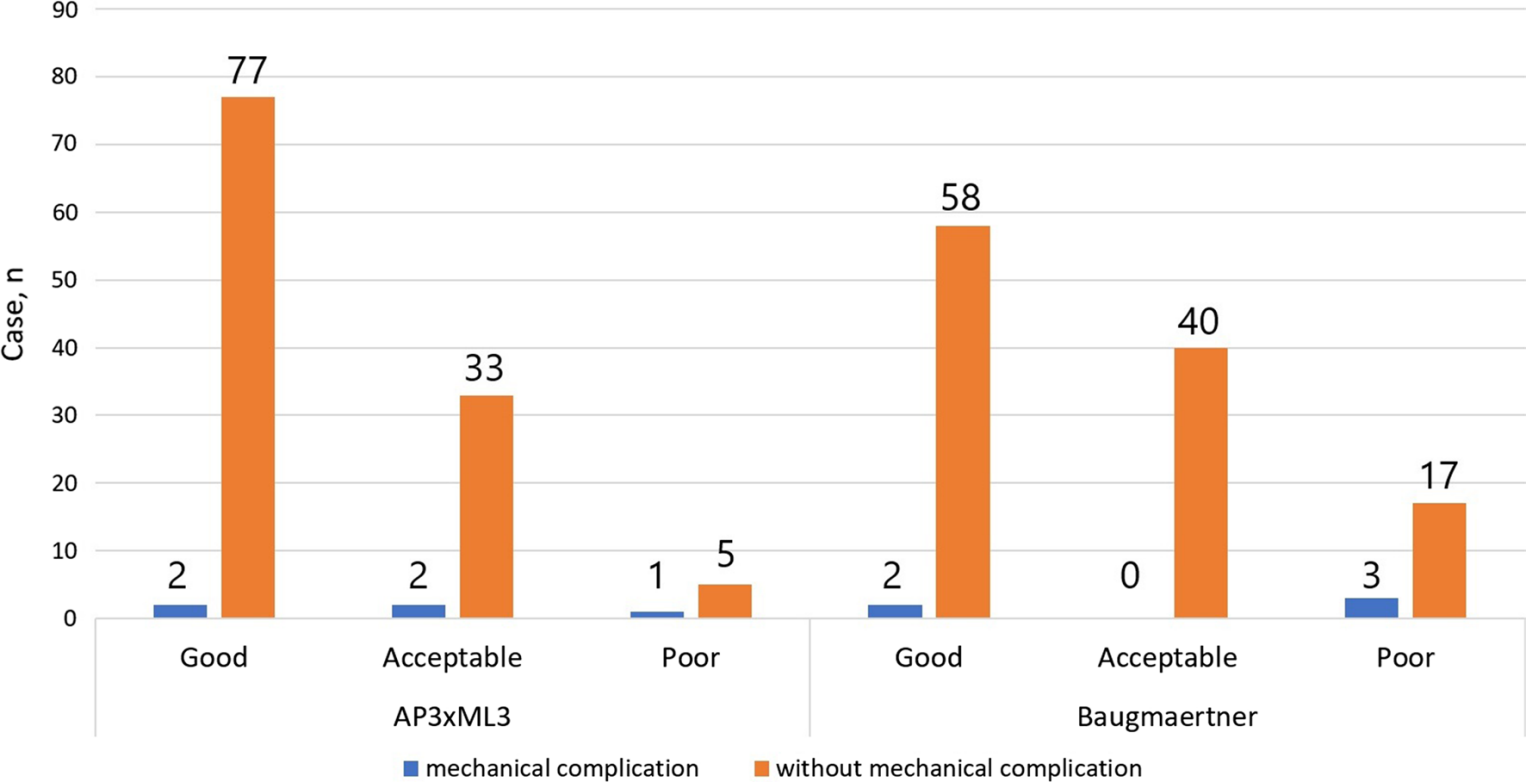
Distribution of outcomes according to AP3 × ML3 classification and Baumgaertner reduction quality criteria AP3 × ML3, AP3 × ML3 classification; Baumgaertner, Baumgaertner reduction quality criteria

Mechanical complications occurred for all levels of reduction quality under each classification, except for the acceptable of the Baumgaertner criteria (Fig. 2). The incidence of mechanical complications according to whether the reduction quality was good, acceptable, or poor was 2.5%, 5.7%, and 16.6% for the AP3 × ML3 classification and 3.3%, 0%, and 15.0% for the Baumgaertner criteria. There was no statistically significant difference in the incidence of mechanical complications among cases classified as poor between the two classifications (*p* = 0.675).

Logistic regression analysis of factors potentially associated with mechanical complications identified poor positioning of the lag screw to be a poor prognostic factor (Table 5).

**Table 5:** Results of logistic regression analysis

Factor	Odds ratio	95% CI	*p* value
Male sex	0.11	0.003–2.99	0.19
Poor reduction according to AP3 × ML3	1.81	0.001–2723.68	0.87
Poor reduction according to Baumgaertner	2.06	0.002–1726.93	0.83
Poor lag screw position	92.03	4.32–1959.65	**0.003**
AO/OTA-A2	2.45	0.13–44.68	0.55

Bold typeface indicates statistically significant results (*p*< 0.05)

*AP3 × ML3* AP3 × ML3 classification, *Baumgaertner* Baumgaertner reduction quality criteria, *AO/OTA* AO Foundation/Orthopedic Trauma Association

## Discussion

The most important finding of this study was that the AP3 × ML3 classification was useful, easy to use, and reliable compared with the Baumgaertner reduction quality criteria.

Fukuda et al. [11] stressed the importance of PMCS when they first described the AP3 × ML3 classification. Kawamura et al. [10] confirmed the stability of the extramedullary reduction pattern when mimicking the concept of PMCS in their biomechanical study. Effective bone-on-bone contact is desirable, and the intramedullary type in the lateral view was reported to be a reduction that should be avoided due to the larger sliding distance of the proximal fragments [16, 17]. In the present study, mechanical complications occurred at a higher rate in the group with poor reduction according to the AP3 × ML3 classification, namely the AP lateral type and the ML intramedullary type. These reduction positions were described by Mao et al. as providing negative medial cortical support [4]. Surgeons should use the AP3 × ML3 classification or the Baumgaertner criteria to achieve a good reduction.

The disadvantage of the Baumgaertner criteria is that they require measurement of the neck–shaft angle and distance. There has been some concern about how accurate the values of 4 mm and 20° are when ethnic and/or sex differences are taken into account [18]. The Chang reduction quality criteria, which are a modified version of the Baumgaertner criteria and based on the concept of PMCS, have recently been reported [2, 4]. The Chang criteria have been found to be reliable in predicting mechanical complications [4] but still rely on the availability of radiographs to measure and evaluate alignment. However, when using the AP3 × ML3 classification, alignment can be defined simply according to the degree of contact between the opposing cortices. Our finding of better inter-observer reliability for the AP3 × ML3 classification than for the Baumgaertner criteria may reflect the ease to use of this new classification system. However, the reason why the inter-observer reliability of the AP3 × ML3 classification was moderate could be that the description of the degree of contact between the medial cortex of the proximal fragment and that of the distal fragment may be somewhat ambiguous and difficult to accurately assess. These were considered to be issues where there is a trade-off with ease of use.

Cutout is one of the most commonly reported mechanical complications of surgery for proximal femoral fractures [3]. Bojan et al. [3] underscored the importance of achieving optimal reduction and positioning of the lag screw to reduce the risk of cutout. In this study, screw position was a significant risk factor for cutout. Most of the mechanical complications that occurred were in cases with poorly positioned screws. Several methods for evaluating screw position have been reported [19], but there is no consensus regarding the best one. The tip-apex distance has been used to describe the screw position [20]. Other methods include the 9-segment technique recommended by Cleveland et al. [21]. Although the method developed Gardenbroek et al. [14] was used in the present study, further research is needed to determine which method is the most appropriate.

This study has several limitations. First, it had a retrospective design, which introduces potential selection bias. In addition, the average time of follow-up was relatively short. It was possible that complications were underestimated. Second, several types of cephalomedullary nails were used. The Gamma3 and InterTAN have been widely used, and there are no reports of a statistically significant difference in clinical outcome between these two types of nails [22]. Similarly favorable outcomes have been reported for the Targon PFT [23]. The Unicorn plus is a dual lag screw cephalomedullary nail used in Japan. According to preliminary reports, the clinical outcomes for the Unicorn plus are non-inferior to those for other types of cephalomedullary nail. Third, we did not evaluate all potential risk factors, such as the bone quality and density in each patient, differences in cephalomedullary nail length, possible toggle movement due to mismatch with the wide proximal canal diameter, and the presence of fractures in the proximal lateral wall [24]. Fourth, the study was conducted at two separate institutions. However, the two surgery centers worked closely together, and all the operations were performed by experienced surgeons, so it is unlikely that there were any marked differences between centers that would have affected the postoperative outcomes. Furthermore, the rate of mechanical complications was comparable between the two institutions. Finally, the sample size was relatively small. In this study, no statistically significant difference was observed in the incidence of mechanical complications among cases classified as poor between the two classifications. Although an increase in the number of cases may lead to statistically significant differences, the purpose of this study was to evaluate the validity of the AP3 × ML3 classification. Further studies are warranted.

## Conclusion

The AP3 × ML3 classification was useful, easy to use, and reliable compared with the Baumgaertner reduction quality criteria.

## Data Availability

The datasets used and/or analyzed during the present study are available from the corresponding author on reasonable request.

## Abbreviations

AP: Anteroposterior
ML: Mediolateral
PMCS: Positive medial cortical support
AO/OTA: AO Foundation/Orthopedic Trauma Association
ICCs: Intraclass correlation coefficients
